# Rationality in Joint Action: Maximizing Coefficiency in Coordination

**DOI:** 10.1177/0956797619842550

**Published:** 2019-05-14

**Authors:** Georgina Török, Barbara Pomiechowska, Gergely Csibra, Natalie Sebanz

**Affiliations:** 1 Department of Cognitive Science, Central European University; 2 Department of Psychological Sciences, Birkbeck, University of London

**Keywords:** social cognition, joint action, coordination, efficiency, rationality, decision making, cooperation, open data

## Abstract

When people perform simple actions, they often behave efficiently, minimizing the costs of movement for the expected benefit. The present study addressed the question of whether this efficiency scales up to dyads working together to achieve a shared goal: Do people act efficiently as a group (i.e., coefficiently), or do they minimize their own or their partner’s individual costs even if this increases the overall cost for the group? We devised a novel, touch-screen-based, sequential object-transfer task to measure how people choose between different paths to coordinate with a partner. Across multiple experiments, we found that participants did not simply minimize their own or their partner’s movement costs but made coefficient decisions about paths, which ensured that the aggregate costs of movement for the dyad were minimized. These results suggest that people are able and motivated to make coefficient, collectively rational decisions when acting together.

People tend to act efficiently when they aim to achieve a goal. For example, on a shopping visit to a mall, shoppers keep to a minimum the walking distance covered between shops of interest (Gärling & Gärling, 1988) to get what they need with the least effort. Motor planning of everyday gestures and movements, such as pointing and grasping, follows the same principle. People move with minimum effort when pointing (Lyons, Hansen, Hurding, & Elliott, 2006), and they guide the movement of their hand to ensure a stable grasp at first contact and to minimize postcontact adjustments (Christopoulos & Schrater, 2009). Furthermore, people sometimes adopt uncomfortable hand positions when these are helpful to continue their action after retrieving an object, suggesting that they plan actions with the total expected effort in mind (Cohen & Rosenbaum, 2004). The motor system often performs comparably with an optimal decision maker (Wolpert & Landy, 2012), selecting the most beneficial solutions in the given circumstances.

How do people achieve efficiency when they work together? Joint actions are aimed at accomplishing shared goals and require coordination between two or more partners (Butterfill, 2017; Sebanz, Bekkering, & Knoblich, 2006). If each interaction partner were to maximize the efficiency of his or her individual actions, this could lead to suboptimal joint performance or a failure to coordinate. Imagine that two friends spot each other from the two ends of a park and would like to sit down for a chat. If each of them walked to the bench closest to her, minimizing her individual cost in terms of walking distance, they may end up sitting on different benches. Sharing the benefits of achieving a joint goal may demand that the actors share the costs as well. Importantly, there are multiple ways to do so, depending on whose costs they want to minimize. How do people distribute the costs of joint actions?

Accounts of team reasoning have proposed that people maximize the aggregate benefits and minimize the aggregate costs of the group (Gilbert, 1987; Hurley, 2005; Sugden, 2000), and empirical evidence for these claims has been provided through interactive economic games (e.g., Colman, Pulford, & Rose, 2008). Minimizing aggregate, rather than individual, costs of an action for a fixed benefit entails aiming for “coefficiency” rather than individual efficiency.

Recent studies have shown that people facilitate their partner’s performance by reducing the partner’s costs. In tasks in which participants handed over objects to another person, they adjusted their own actions to reduce the effort of the partner who concluded the action sequence. They rotated objects (Constable et al., 2016; Dötsch & Schubö, 2015; Ray & Welsh, 2011), selected particular grasp types (Scharoun, Scanlan, & Bryden, 2016), chose appropriate grasp locations on an object (Meyer, van der Wel, & Hunnius, 2013), and handed over objects at spatial locations that made it easier for the partner to finish the task (Gonzalez, Studenka, Glazebrook, & Lyons, 2011; Ray, de Grosbois, & Welsh, 2017; Scharoun, Mintz, Glazebrook, Roy, & Gonzalez, 2017).

Further evidence for spontaneous sharing of effort comes from an observational study that investigated how people hold doors open for others behind them (Santamaria & Rosenbaum, 2011). The closer a follower, the more likely people were to hold open the door; the door was held open for longer when two people followed than when only one followed; and when the door was held open, followers sped up to reach it. Although these findings are generally in line with the idea that people are sensitive to aggregate group effort, they do not clarify why. People might be helping their partners; that is, people might incur extra costs to reduce the partner’s costs. Alternatively, people might act coefficiently, which differs from altruistic behavior in that the person incurring costs aims to minimize aggregate group costs rather than the coactor’s costs.

Numerous real-world situations, from cooking together to dividing paperwork to raising children, require partners to coordinate and invest efforts to achieve shared goals. To shed light on the question of how people distribute costs of joint activities, we pitted coefficiency against helping by investigating the coefficiency of joint action planning in a context in which individual and aggregate costs of two actors were systematically manipulated. We operationalized action cost as proportional to path length in a task that required participants to move objects from one location to another. In this context, maximizing efficiency amounted to taking the shortest available path to a goal, given environmental constraints. The joint version of the task involved passing an object to a partner at one of two transfer locations (see Fig. 1). The person passing the object could optimize either his or her own efficiency, choosing the shortest subpath to a transfer location, or the total executed path length of the dyad. In some trials, taking the shorter subpath from an individual perspective resulted in an overall shorter path for the dyad (congruent trials). In other trials, taking the shorter subpath from an individual perspective corresponded to an overall longer path for the dyad (incongruent trials). In further trials, the two paths were equal in length from a dyadic point of view (neutral trials) but differed in terms of the relative subpath lengths of the two actors. If people maximize coefficiency, they should specifically incur higher individual costs on incongruent trials to reduce joint costs. If they maximize individual efficiency, they should consistently take the shorter subpath, regardless of the overall joint costs. Finally, if people are being helpful, they should act to minimize their partner’s individual cost, either when this does not impair coefficiency (on neutral trials) or when it does (taking the longer subpath on congruent trials, which would minimize the subpath for the partner but increase the overall path length).

**Fig. 1.**
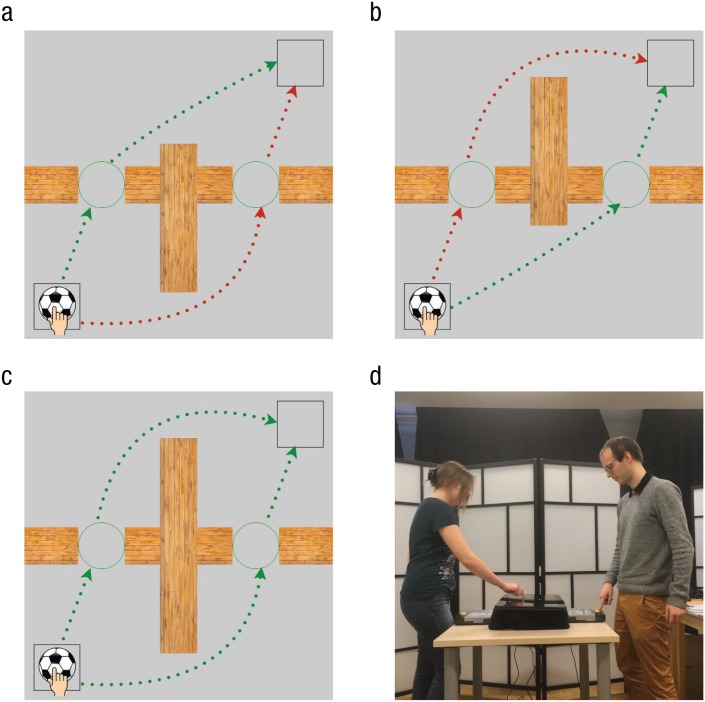
Example experimental layouts and a photo of the experimental setup. In all conditions, participants’ task was to move the ball from the starting location to the goal location (both indicated by squares) through one of the circles that marked the possible subgoals. In the congruent condition (a), the shorter subpath from an individual perspective resulted in an overall shorter path for the dyad; in the incongruent condition (b), the shorter subpath from an individual perspective corresponded to an overall longer path for the dyad; and in the neutral condition (c), the two paths were equal in length from a dyadic point of view. Efficient total paths (Experiment 1) and coefficient total paths (Experiment 2) are colored green, and subefficient total paths are colored red (the arrows in the figure are for illustration). The experimental setup and the actors’ positions in Experiment 2 are shown in (d).

To ensure that the costs associated with the different paths were perceivable and that our task afforded cost optimization, we first ran an individual version, in which single participants performed both steps of the object-transfer task (Experiment 1). We then investigated joint performance (Experiment 2).

## Experiment 1: Individual Efficiency

This experiment tested whether people maximize efficiency of individually executed action sequences. We gave participants a choice between two paths along which they could move a ball: a shorter path and a longer path. If people act efficiently, they should consistently select the shorter path. The exact proportion of efficient choices might be influenced by the degree of asymmetry between available paths: The larger the length difference between the paths, the more sensitive people might be to cost differences. To test this, we manipulated the difference in length between paths.

### Method

#### Participants

On the basis of a power analysis in G*Power 3 (Faul, Erdfelder, Lang, & Buchner, 2007), we estimated that a sample size of 24 would be needed to provide 80% statistical power to achieve a medium effect size (*d* = 0.6) on binary choices using a one-sample *t* test against a 50% chance level and an alpha of .05. The participants were recruited through Central European University’s Research Participation System (developed by SONA Systems; https://www.sona-systems.com/default.aspx) and a student job agency. Participants gave informed consent and received vouchers in exchange for their participation. The study was approved by the United Ethical Review Committee for Research in Psychology in Hungary. Twenty-seven right-handed participants took part in the experiment. We analyzed the data of 24 participants (7 male; age: *M* = 25.1 years, *SD* = 3.54). We excluded 3 participants because of a computing error (*n* = 1) or an experimenter error (*n* = 2).

#### Apparatus

The task was performed on a touch-screen monitor (Elo Touch Solutions, 2201L, 22-in., 1,920 × 1,080 pixels resolution, 60 Hz) lying flat (screen up) on a table and connected to an Apple iMac computer. Stimulus presentation and data recording were controlled by a script using the Psychophysics Toolbox (Brainard, 1997; Kleiner, Brainard, & Pelli, 2007; Pelli, 1997) in MATLAB (The MathWorks, Natick, MA). A response box (The Black Box ToolKit, Sheffield, UK) was used to control trial onset.

#### Stimuli

On each trial, the display consisted of the following elements: an image of a soccer ball, a starting position, a goal position, and obstacles (see Fig. 1). The starting and goal positions, marked by squares, were located in diagonally opposite corners of the screen. The ball was initially placed at the starting position. Obstacles consisted of (a) a wall placed in the middle of the screen that separated the starting position and the goal position, with two gaps through which to pass the ball (marked by circles), and (b) two additional barriers, located perpendicular to the wall on each of its sides (see Fig. 1). One barrier had a fixed (maximal) length of 1 unit and was located either on the side of the screen nearer to the participant or on the side farther from the participant. The size of the barrier on the other side of the wall varied between 0 (no barrier) and the maximal length in 0.25-unit steps, resulting in five distributions of barrier lengths: 1–0, 1–0.25, 1–0.5, 1–0.75, and 1–1. These combinations of barrier lengths on each side of the screen provided participants with different degrees of asymmetry between the costs of moving to the gap closer to or farther from their starting positions. For example, a 0.75-unit-long barrier on the participant’s side resulted in a much longer subpath to the gap farther away from the participant than the subpath to the gap closer to the participant. In contrast, a 0-unit-long barrier (i.e., no barrier perpendicular to the wall between the two sides of the screen) imposed the least difference between the short and long subpath options for the participant. The wall with the circled gaps in it was either parallel or perpendicular to the longer side of the touch screen, with half of the trials displaying a horizontal wall and the other half displaying a vertical wall.

#### Procedure

The starting position of the soccer ball was always on the same side of the table at which participants were standing. They were instructed to pull the ball with their finger from the starting position to the goal position through one of the gaps in the wall. The movement of the ball was blocked if any pixel of the ball image overlapped with a pixel of the displayed walls, barriers, and screen boundaries—an event we referred to as a collision. All instances of such collisions were registered and signaled to the participants by an audio sound bite. Participants were instructed to complete the task as accurately as possible, that is, with the fewest collisions with the obstacles.

The participants were instructed to keep their dominant hand on the response box at the beginning of each trial. The box was placed perpendicular to the touch screen along the middle of the screen’s longer side. This ensured that the key on the box was equidistant from the two potential starting positions at the left and right corners of the screen. When participants started pressing the key on the response box, the layout was presented without the ball. After 1,500 ms, the ball appeared in the starting position, which prompted the participants to release the key and start moving the ball. When the ball arrived in the circle at one of the gaps, the subgoal was completed. To indicate this, the background of the circle was highlighted, the movement of the ball was blocked, and participants had to briefly release it before they could resume dragging it farther. As soon as the ball arrived at the goal area, a short auditory signal marked the completion of the trial.

Before the experiment, participants completed a brief practice session of 10 trials to familiarize themselves with the use of the touch screen, the response box, and the screen layouts. They then completed 80 trials: 32 congruent trials, 32 incongruent trials, and 16 neutral trials. In congruent trials, passing the ball through the gap closer to the starting position (i.e., taking the short subpath to the subgoal of passing through the wall) coincided with taking the overall shorter path to the goal location (see Fig. 1a). In incongruent trials, the short subpath was part of the longer total path to the goal location (see Fig. 1b). Neutral trials were symmetric in terms of total path lengths (see Fig. 1c). The length of the shorter barrier in the nonneutral trials, the orientation of the layout (horizontal or vertical wall), and the starting positions (left side or right side) were fully counterbalanced. The order of trials was randomly determined. Participants completed the task in an average of 14.22 min (*SD* = 2.11). At the end, participants filled out a short questionnaire on what they thought to be the purpose of the experiment.

#### Data analysis

The primary dependent variable was the proportion of efficient path choices, that is, the proportion of trials in which the participants chose the shorter total path between the starting and goal locations. Choice-proportion data were not normally distributed; therefore, all statistical analyses were performed on arcsine-transformed proportion data. All comparisons were conducted in JASP (JASP Team, 2018) using Wilcoxon signed-rank tests (two tailed), unless otherwise noted. We report *V* statistics for the Wilcoxon tests, as well as matched-pairs rank-biserial correlation coefficients (*r*s), both provided by JASP. The *V* statistic corresponds to the sum of ranks assigned to positive-signed differences between the two tested paired samples and represents the value to be compared with those found in tables for the Wilcoxon test. The matched-pairs rank-biserial correlation coefficient represents the effect size of the difference between the paired variables. The lower the value of *r*, the lower the difference between positive and negative rank sums and, therefore, the smaller the size of the effect that rendered the two paired samples different.

To assess whether choosing the efficient option resulted in faster or more accurate performance, we also analyzed the mean number of collisions per trial (to estimate accuracy) and total trial durations (to estimate average speed) according to the choices that actors made. Duration measurements were log-transformed for analyses. For ease of reading, the text and figures report untransformed summary statistics. In all cases, confidence intervals (CIs) are reported for the difference between the values analyzed in the corresponding statistics.

### Results

#### Proportion of efficient choices

**Fig. 2.**
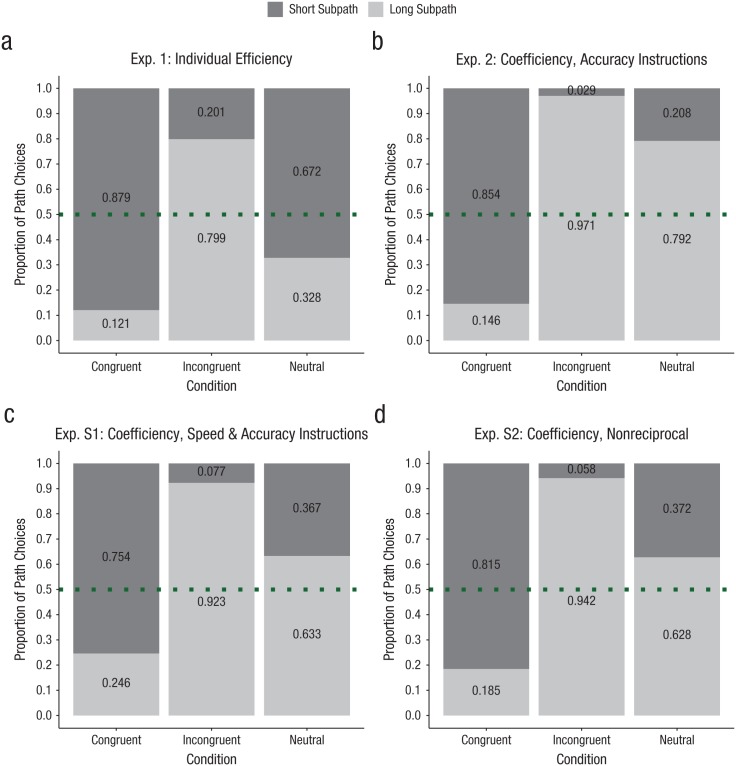
Mean raw proportion of short- and long-subpath choices in each of the three conditions of (a) Experiment 1, (b) Experiment 2, and two additional joint-action experiments: (c) Experiment S1 and (d) Experiment S2 (*N*s = 24). Experiments 2 and S1 differed only with regard to the task instructions, whereas in Experiment S2, only one of two partners made choices. For further details and results of Experiments S1 and S2, see the Supplemental Material available online. Efficient choices (Experiment 1) and coefficient choices (Experiments 2, S1, and S2) were taking the short subpath in the congruent condition and the long subpath in the incongruent condition. Dotted lines indicate the chance level (.5) of choice proportion.

Participants tended to minimize the total path length. They transferred the object in an efficient manner, that is, through the gap that was closer to them in the congruent trials (*M* = .88, *SD* = .21) and through the farther gap in the incongruent trials (*M* = .80, *SD* = .28; see Fig. 2a). Efficient-choice ratios were significantly different from chance—congruent: *V* = 294, *p* < .001, *r* = .96, 95% CI for the difference between the proportion of efficient path choices and chance level (arcsine-transformed chance level of .5 = .7854) = [1.21, 1.48]; incongruent: *V* = 253, *p* < .001, *r* = .69, 95% CI = [1.06, 1.42]. Efficiency did not differ between the congruent and incongruent trials, as suggested by a paired-samples comparison between the ratio of efficient choices in the two conditions (*V* = 116, *p* = .065, *r* = −.23, 95% CI = [−0.02, 0.26]; see Fig. 2a). In the neutral trials, participants tended to choose the closer gap (*M* = .67, *SD* = .18; *V* = 234.5, *p* < .001, *r* = .56, 95% CI = [0.89, 1.09]). Paired-samples comparisons to matching subpath choices in the neutral condition showed a significant increase in the proportion of closer-gap choices in congruent trials (*V* = 239, *p* < .001, *r* = .59, 95% CI = [0.26, 0.52]) and a significant decrease in incongruent trials (*V* = 4, *p* < .001, *r* = −.97, 95% CI = [−0.80, −0.45]). That is, in asymmetric trials, participants shifted their decision toward the more efficient choice, compared with the neutral trials.

**Fig. 3.**
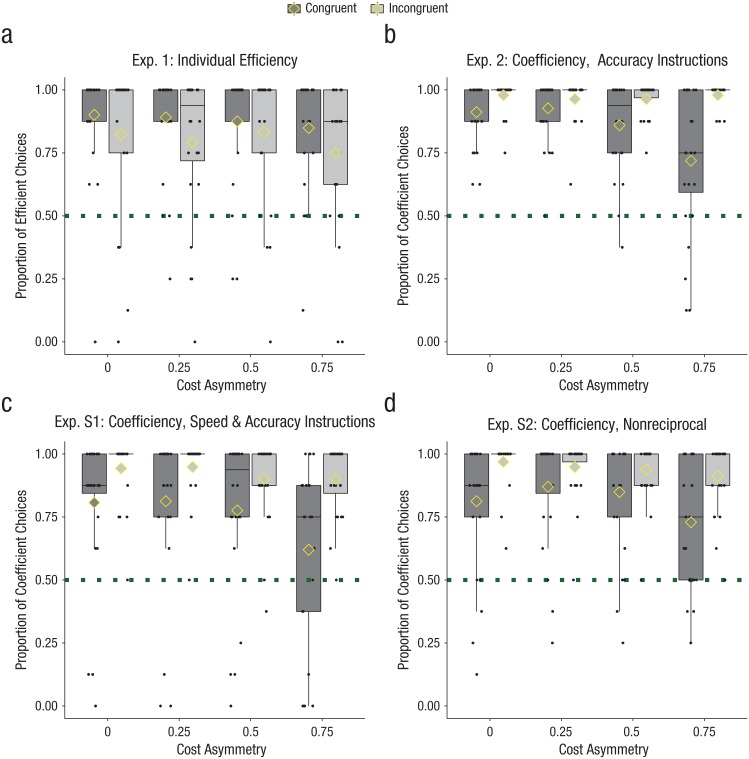
Raw proportion of efficient path choices for each cost asymmetry in the congruent and incongruent conditions of (a) Experiment 1, (b) Experiment 2, and two additional joint-action experiments: (c) Experiment S1 and (d) Experiment S2 (*N*s = 24). For details and further results of Experiments S1 and S2, see the Supplemental Material available online. Each black dot represents a participant’s efficient-choice ratio in the given condition. In each data bar, horizontal lines indicate medians, and diamonds indicate mean efficient-choice proportions. Whiskers extend 1.5 times the interquartile range. The dotted lines indicate the chance level (.5) of choice proportion.

We analyzed whether the size of the difference in length between the path options had an effect on participants’ efficient path choices using a 4 × 2 repeated measures ANOVA with cost asymmetry (0-, 0.25-, 0.5-, and 0.75-unit lengths of the central barrier on one side of the screen vs. a 1-unit-long barrier on the other side) and condition (congruent vs. incongruent) as factors (see Fig. 3a). This analysis yielded a statistically significant main effect of cost asymmetry, *F*(3, 69) = 4.83, *p* = .004, η^2^ = .17. Post hoc tests revealed that this effect was due to a decrease in the proportion of efficient choices in trials with a 0.75-unit-long barrier compared with shorter lengths—a post hoc Bonferroni-corrected *t* test comparing 0.75 with 0 found a statistically significant difference in the proportion of efficient choices, *t*(23) = 3.20, *p* = .024, *d* = 0.65, 95% CI = [.04, .20]—whereas comparisons with 0.25- and 0.5-unit lengths, respectively, found only tendencies for higher efficiency ratios in trials with the shorter barriers: *t*(23) = 2.80, *p* = .062, *d* = 0.57, 95% CI = [.02, .15]; *t*(23) = 2.78, *p* = .064, *d* = 0.57, 95% CI = [.03, .20]. Neither the main effect of condition, *F*(1, 23) = 3.46, *p* = .076, η^2^ = .13, nor the interaction between cost asymmetry and condition was statistically significant, *F*(3, 69) = 1.48, *p* = .227, η^2^ = .06.

#### The effects of choices on performance

To test whether choosing the efficient path improved the accuracy and the speed of object transfer, we analyzed the mean frequency of collisions per trial and mean trial duration according to the decisions made. When we considered that participants exhibited a strong tendency to make efficient choices throughout the experiment, we found that the number of subefficient choices was much lower than that of efficient choices. Five participants did not make any subefficient choices. On average, participants completed the trials with the same level of accuracy when making efficient decisions (*n* = 24, *M* = .10, *SD* = .07) as when choosing the subefficient path (*n* = 19, *M* = .15, *SD* = .20), *t*(18) = 1.10, *p* = .286, *d* = 0.25, 95% CI for the mean difference = [−.05, .15]. However, a paired-samples *t* test on mean trial durations demonstrated that the participants completed the task more slowly when making subefficient choices (*n* = 19, *M* = 7.88 s, *SD* = 1.83) than efficient choices (*n* = 24, *M* = 6.06 s, *SD* = 1.02), *t*(18) = 9.72, *p* < .001, *d* = 2.23, 95% CI of difference on log-transformed data = [0.09, 0.14].

#### Exploratory analyses

To address the question of whether participants had become more efficient over the course of the task, we compared the proportion of efficient choices in the first half (Block 1, 40 trials) with those in the second half (Block 2, 40 trials) of the experiment using Wilcoxon signed-rank tests. We found that, in the incongruent condition, the proportion of efficient choices increased between Blocks 1 and 2 (Block 1: *M* = .75, *SD* = .33; Block 2: *M* = .85, *SD* = .24; *V* = 5, *p* < .001, *r* = −.97, 95% CI = [−0.32, −0.13]). We observed no such increase in the congruent condition (Block 1: *M* = .86, *SD* = .24; Block 2: *M* = .90, *SD* = .19; *V* = 36, *p* = .315, *r* = −.76, 95% CI = [−0.27, 0.07]). However, one-sample comparisons with chance also suggested that in Block 1, the ratios of efficient path choices were already significantly higher than chance level, regardless of condition—congruent: *V* = 289, *p* < .001, *r* = .92, 95% CI for the difference between the proportion of efficient choices and chance (arcsine-transformed chance level of .5 = .7854) = [.36, .79]; incongruent: *V* = 237, *p* = .013, *r* = .58, 95% CI = [0.15, 0.58].

In short, we found that participants in Experiment 1 already made efficient choices in the first half of the experiment. However, on trials in which taking the longer subpath first was the efficient decision (incongruent condition), participants chose it more frequently over time, suggesting that practice had some effect on making efficient choices.

### Discussion

Participants acted efficiently, predominantly choosing the shorter total path to transfer the object. This was more pronounced for layouts in which the difference in path length was larger, resulting in higher cost asymmetry. Choosing the shorter path resulted in shorter trial-completion times. The tendency to choose the gap closer to the starting position in neutral trials indicates that participants may have prioritized completing the first subgoal (cf. Rosenbaum, Gong, & Potts, 2014).

## Experiment 2: Coefficiency

To test the hypothesis that people maximize the coefficiency of joint actions, we had pairs of participants perform the task together as a sequentially distributed joint action. The coefficiency hypothesis predicts that the actor initiating the joint action should choose the subpath that results in the shortest path for the dyad rather than minimizing his or her own or his or her partner’s movement distance.

### Method

#### Participants

Target sample size was determined in the same way as for Experiment 1, by conducting a power analysis in G*Power 3 (Faul et al., 2007). It was estimated that a sample size of 24 would be needed to provide 80% statistical power. Twenty-eight right-handed participants took part in Experiment 2. We excluded two pairs from data analysis because of a computing error (*n* = 1) and failure to understand the instructions (*n* = 1). We report the results of 12 dyads (4 mixed-gender and 4 female dyads; *N* = 24; 12 male; age: *M* = 25.4 years, *SD* = 4.14). In all joint experiments (including Experiments S1 and S2, detailed in the Supplemental Material available online), we excluded dyads’ data if they had previously known each other to prevent any confound related to familiarity. For Experiment 2, we did not happen to recruit participant pairs who were familiar with each other.

#### Apparatus

We used the same apparatus as in Experiment 1. Using the Psychophysics Toolbox (Brainard, 1997; Kleiner et al., 2007; Pelli, 1997) in MATLAB, we controlled stimulus presentation and data recording with a script of the task adapted for dyads. Two response boxes (The Black Box ToolKit) were used, one for each participant.

#### Stimuli and task

Experiment 2 employed the same stimuli and task as Experiment 1, with the difference that both members of the dyad had to act jointly to transfer the ball from the starting location to the goal location: One participant moved the ball to the subgoal location (i.e., one of the two gaps in the wall), and the other moved it from there to the goal location. Participants took turns completing each part of the action sequence in a trial. The subgoal of transporting the ball to a gap in the middle of the screen was assigned to the decision-making participant, who acted first on the given trial (Actor 1). After Actor 1 handed over the ball to his or her partner (Actor 2), he or she moved it from the gap to the goal location and thus completed the task. The role of Actor 1 was randomly assigned throughout the task in each trial, and both participants acted as Actor 1 and Actor 2 an equal number of times.

#### Design

This experiment had the same design as Experiment 1. The primary dependent variable was Actor 1’s choice of subpath to a subgoal, that is, to the gap where he or she would transfer the ball to his or her partner, Actor 2. Accordingly, the main factor that we manipulated was whether choosing the gap that offered the shorter subpath to achieve Actor 1’s goal of passing the ball to his or her partner resulted in a shorter total path for the dyad. When the central barrier was longer on Actor 1’s side than the one on the other side (see Fig. 1a), maximizing either individual efficiency or coefficiency required Actor 1 to choose the closer gap (congruent trials). When the central barrier was longer on the opposite side (see Fig. 1b), maximizing coefficiency required Actor 1 to opt for the farther gap, and maximizing his or her individual efficiency meant choosing the closer gap (incongruent trials). When the barrier lengths on the two sides were equal (see Fig. 1c), either choice resulted in the same total path length (neutral trials).

Congruent and incongruent trials had the same levels of asymmetry between path lengths as in Experiment 1 (see different barrier lengths of the cost-asymmetry factor). The list of trials from Experiment 1 was duplicated so that each participant completed the 80 trials used in Experiment 1. Trial order was random.

#### Procedure

Participants faced one another, standing on the two opposite sides of the touch screen lying face up on a table, and had full visual access to what their partner was doing (see Fig. 1d). Because we used a turn-taking task, only the acting player was in control of the ball. In the meantime, the partner had to keep a key pressed on the response box in front of him or her. Participants were instructed to finish each trial as accurately as possible while minimizing collisions and to avoid communicating with one another during the task. The instructions also emphasized the shared goal of moving the ball from one side of the screen to the other. Participants first completed a brief practice session of 10 trials, followed by the main experimental task. Finally, they filled out a short questionnaire regarding what they thought to be the experiment’s purpose and how much they liked their partner using a 7-point Likert-type scale (1 = *not at all*, 7 = *very much*).

At the beginning of each trial, when both actors pressed and held down the keys on their respective response boxes, they saw the layout of the game on screen, which displayed their starting squares without the ball image. After 1,500 ms, the ball appeared in one of the squares. The actor with the object on his or her side (Actor 1) moved first and chose a transfer point to pass the ball over to his or her partner through one of the two circled gaps between the walls (see Figs. 1a–1c). When the ball was fully inside the circle, the background of the circle was highlighted, any further movement of the ball by Actor 1 was blocked, and he or she had to press the response key again. Actor 2 then moved his or her hand from the respective response box key to the ball and dragged it back to the goal location on his or her side.

Two movement trajectories were registered: Actor 1’s move to the gap from the starting location, and Actor 2’s move from the gap to the goal location. A trial was complete when Actor 2 took the ball back to the home square (the goal location). No feedback was provided about speed or accuracy of performance. Each dyad completed the task in their own time. Participants completed the task in an average of 21.49 min (*SD* = 2.89).

#### Data analysis

Data transformations and analyses were identical to those in Experiment 1. The primary dependent measure was the proportion of Actor 1’s coefficient choices, that is, the shorter subpath in the congruent condition and the longer subpath in the incongruent condition. In all cases, CIs are reported for the difference between the values analyzed in the corresponding statistics.

### Results

#### Proportion of coefficient choices

Participants opted for subpaths that maximized the coefficiency of the dyad (see Fig. 2b): One-sample Wilcoxon tests indicated that in congruent trials, participants passed the ball through the gap closer to them significantly more often than chance (*M* = .85, *SD* = .14), *V* = 300, *p* < .001, *r* = 1.00, 95% CI for the difference between the proportion of coefficient choices and chance (arcsine-transformed chance level = .7854) = [1.13, 1.36], whereas in incongruent trials, they chose the gap farther away (*M* = 0.97, *SD* = 0.04), *V* = 300, *p* < .001, *r* = 1.00, 95% CI = [1.39, 1.48]. In neutral trials, participants were significantly more likely to choose the longer subpath on their side than the shorter one (*M* = 0.79, *SD* = 0.23), *V* = 277, *p* < .001, *r* = .85, 95% CI = [1.05, 1.31]. Paired-samples comparisons confirmed that the proportions of coefficient choices were higher in both the congruent trials, *V* = 300, *p* < .001, *r* = 1.00, 95% CI = [0.68, 1.00], and the incongruent trials, *V* = 210, *p* < .001, *r* = .40, 95% CI = [0.25, 0.42], than the proportions of the short and long subpath choices in the neutral trials, respectively. Furthermore, we found that 3 participants never chose subpaths that were subefficient from the dyad’s perspective.

A paired-samples comparison between the ratio of short subpath choices in congruent trials and long subpath choices in incongruent trials found that the ratio of coefficient choices in the incongruent trials was significantly higher than in the congruent trials (*V* = 173.5, *p* = .002, *r* = .16, 95% CI = [0.13, 0.39]; see Fig. 2b). Participants made more coefficient path choices when this meant reducing the effort of their partner than otherwise.

Efficient decisions were compared among different degrees of cost asymmetry in a 4 (cost asymmetry) × 2 (condition) repeated measures ANOVA on the ratios of short and long coefficient subpath choices. We found that the participants chose the coefficient paths more often in incongruent than in congruent trials—main effect of condition, *F*(1, 23) = 17.13, *p* < .001, η^2^ = .43 (see Fig. 3b). The participants chose coefficient paths more frequently in trials with layouts with shorter barriers than in ones with longer barriers, as suggested by a statistically significant main effect of cost asymmetry, *F*(3, 69) = 6.30, *p* < .001, η^2^ = .22. Furthermore, we found a statistically significant Cost Asymmetry × Condition interaction, *F*(3, 69) = 7.48, *p* < .001, η^2^ = .25. This was due to a difference between the sizes of the condition effect on proportions of coefficient choices in trials with different degrees of cost asymmetry. Post hoc Bonferroni-corrected paired-samples *t* tests yielded statistically significant effects of condition on the ratio of coefficient choices in trials with 0-, 0.5-, and 0.75-unit-long barriers, respectively—0 unit: *t*(23) = 2.73, *p* = .048, *d* = 0.56, 95% CI = [0.04, 0.25]; 0.5 unit: *t*(23) = 2.98, *p* = .028, *d* = 0.61, 95% CI = [0.05, 0.30]; 0.75 unit: *t*(23) = 4.74, *p* < .001, *d* = 0.97, 95% CI = [0.23, 0.59]—but not in trials with 0.25-unit-long barriers (*p* = 1.000). We found that for most combinations of barrier lengths, it was true that Actor 1 made more coefficient decisions when coefficiency entailed helping his or her partner by choosing the gap that was farther away (incongruent trials) rather than the gap that was closer (congruent trials).

#### The effect of choices on performance

To test whether Actor 1’s coefficient choices improved the dyad’s performance, we compared the mean frequency of collisions per trial and mean trial duration between trials in which Actor 1 chose the coefficient subpath and those in which Actor 1 chose the subefficient subpath. On average, dyads completed trials with a significantly higher level of accuracy when Actor 1 chose the coefficient subpath, colliding with on-screen walls fewer times (*n* = 24, *M* = .16, *SD* = .10) than when he or she chose the subefficient path (*n* = 21, *M* = .33, *SD* = .42), *t*(20) = 2.18, *p* = .041, *d* = 0.48, 95% CI = [.01, .34]. Although actors were not explicitly instructed to optimize speed, making coefficient decisions also resulted in shorter trial-completion times. Trial duration was significantly longer for subefficient choices (*n* = 21, *M* = 10.7 s, *SD* = 5.62) than for efficient choices (*n* = 24, *M* = 7.55 s, *SD* = 1.02), *t*(20) = 5.85, *p* < .001, *d* = 1.28, 95% CI = [0.08, 0.17].

#### Questionnaires

In the questionnaire addressing the perceived purpose of our study, one third of the participants said they thought the experiment was investigating cooperation (*n* = 7) and helping tendencies (*n* = 8). A minority of the participants made explicit reference to rational decision making or optimization (*n* = 4), finding the shortest path for both players (*n* = 5), and reactivity to a partner’s actions (*n* = 6), and a few people thought that we were looking at the effect of getting tired or being good at perceiving visual differences in distances (*n* = 3).

The ratings of partners were generally high (*Mdn* = 6, *SD* = 0.95). The correlation (Spearman’s ρ) between liking ratings and the arcsine-transformed ratios of coefficient choices was not different from zero in either condition (congruent: ρ = .321, *p* = .126; incongruent: ρ = −.076, *p* = .725).

#### Exploratory analyses

As in Experiment 1, we conducted additional exploratory analyses to address the potential influence of practice on efficient decision making by comparing the proportion of coefficient choices between the first half (Block 1, 80 trials) and the second half (Block 2, 80 trials) of the joint task. Paired-samples Wilcoxon signed-rank tests found that in the congruent condition, the proportion of coefficient choices increased between Blocks 1 and 2 (Block 1: *M* = .82, *SD* = .19; Block 2: *M* = .89, *SD* = .13; *V* = 35.5, *p* = .031, *r* = −.76, 95% CI = [−0.28, −0.01]). No such increase was observed in the incongruent condition (Block 1: *M* = .96, *SD* = .10; Block 2: *M* = .98, *SD* = .04; *V* = 35, *p* = .484, *r* = −.77, 95% CI = [−0.31, 0.24]). Proportions of coefficient choices were already significantly higher than chance in Block 1, regardless of condition (all *p*s < .001).

To investigate potential between-experiment differences in the ratios of efficient (Experiment 1) and coefficient (Experiment 2) choices, we compared the ratios of efficient and coefficient decisions in the congruent and incongruent conditions separately. Mann-Whitney *U* tests with experiment as a factor found no statistically significant difference in the proportion of efficient and coefficient choices in the congruent condition (Experiment 1: *M* = .88, *SD* = .21; Experiment 2: *M* = .85, *SD* = .14; *U* = 351.5, *p* = .184, *r* = .22, 95% CI for the median difference between the two experiments = [−3.49^e–5^, 0.31]). In contrast, in the incongruent condition, dyads in Experiment 2 made a statistically significantly higher proportion of coefficient choices than individuals in Experiment 1 made efficient choices (Experiment 1: *M* = .80, *SD* = .28; Experiment 2: *M* = .97, *SD* = .04; *U* = 168, *p* = .011, *r* = −.42, 95% CI = [−0.31, −9.572^e–6^]). This asymmetric pattern in between-experimental differences suggests that facilitating a partner’s actions in the joint task by taking the longer subpath might have further boosted the ratio of coefficient choices.

### Discussion

When participants had multiple options to plan a movement in a coordination context, they considered not just their own but also their partner’s costs. This was demonstrated by the first actors’ strong tendency to choose the subpath that was more coefficient, whether it resulted in reducing or increasing their partner’s costs. That is, action initiators chose the shorter subpath for themselves and the longer one for their partner in the congruent condition, and they displayed the opposite pattern of choices in the incongruent condition. When coefficiency was unaffected by subpath choices (neutral trials), participants reduced their partner’s costs.

## General Discussion

Our experiments addressed the question of whether people minimize the aggregate costs of actions when cooperating with others to reach a shared goal. We operationalized action costs as path length traveled while moving an object. We found that actors chose to minimize the total path length when offered two path options to complete a movement sequence. In the joint task, these total paths were distributed over coactors, suggesting that participants aimed at maximizing the coefficiency of the dyad. In the individual task, the choices were similar to joint performance, demonstrating efficient planning for the entire action sequence.

The decisions in the dyadic incongruent condition, in which taking a longer subpath to a gap was analogous to reducing the partner’s effort in joint object-manipulation tasks (Dötsch & Schubö, 2015; Meyer et al., 2013), indicated that actors integrated their partner’s effort into their planning and were motivated to reduce their partner’s costs. However, in the congruent condition, participants refrained from reducing their partner’s effort, maximizing the group’s efficiency by forcing partners to move along the longer subpath. The complementary pattern of the two conditions suggests that, in joint-action contexts, people aim at reducing aggregate group costs rather than minimizing the effort of either party. This is in line with Santamaria and Rosenbaum’s (2011) shared-effort model, which postulates that actors coordinate their actions to reduce aggregate costs of a group.

We tested the robustness of coefficiency maximization in two additional experiments (see the Supplemental Material). The results of Experiment 2 were replicated when participants were instructed to complete the task as quickly as possible in addition to being accurate (Experiment S1) and when the identity of the decision maker was fixed to eliminate turn taking of choices (Experiment S2). The latter results indicated that expectation of reciprocity is not necessary for efficient jointaction planning.

Notably, the congruent and incongruent trials induced similar decisions already in the first half of the task in both experiments. This raises the possibility that, in the joint task, decision makers disregarded their partners entirely when planning their actions and considered only the total path options that they could have executed individually. The differential results of the neutral trials, however, provide evidence against this account: When coefficiency did not discriminate between the options, participants reduced their partner’s costs by covering the longer distance (Experiment 2) but were biased in the opposite direction when they acted alone (Experiment 1). Furthermore, in Experiment 1, participants maximized efficiency similarly across conditions, whereas in Experiment 2, they made more coefficient decisions in the incongruent condition than in the congruent condition. Lastly, we observed a higher proportion of coefficient choices in the incongruent condition of Experiment 2 relative to Experiment 1. In other words, actors sacrificed the efficiency of their initial act more when this choice reduced the partner’s effort than when it increased the partner’s costs or when they performed the task alone. These findings suggest that the participants planned the joint-action sequences with their partners in mind, possibly even signaling cooperative attitudes by taking over effort from them when this decision did not compromise coefficiency.

Future experiments should address the mechanism underlying coefficiency maximization in more detail. Candidate mechanisms for such decision making include a rational calculus of joint costs, which sums agent-specific individual costs, along with the use of heuristics, such as simulating entire action sequences to be performed by the individual alone. Beyond specifying the mechanism, a model of rational joint action planning will need to explore the boundary conditions of coefficiency maximization. In the present study, we focused on path length, but actions may similarly be optimized for exerted effort, in which case movement curvature could also be considered. Finally, joint optimization could be modulated by benefit sharing and asymmetries in competence or in access to information.

## Supplemental Material

TorokSupplementalMaterial_rev – Supplemental material for Rationality in Joint Action: Maximizing Coefficiency in Coordination Click here for additional data file. Supplemental material, TorokSupplementalMaterial_rev for Rationality in Joint Action: Maximizing Coefficiency in Coordination by Georgina Török, Barbara Pomiechowska, Gergely Csibra and Natalie Sebanz in Psychological Science

## Supplemental Material

Torok_OpenPracticesDisclosure_rev – Supplemental material for Rationality in Joint Action: Maximizing Coefficiency in Coordination Click here for additional data file. Supplemental material, Torok_OpenPracticesDisclosure_rev for Rationality in Joint Action: Maximizing Coefficiency in Coordination by Georgina Török, Barbara Pomiechowska, Gergely Csibra and Natalie Sebanz in Psychological Science
