# Supplementary material for: Rationality in Joint Action: Maximizing Coefficiency in Coordination
Source: Psychol Sci. 2019 May 14;30(6):930–41. doi: 10.1177/0956797619842550 (PMC6560520; doi:10.1177/0956797619842550)
Supplement: TorokSupplementalMaterial_rev – Supplemental material for Rationality in Joint Action: Maximizing Coefficiency in Coordination [file TorokSupplementalMaterial_rev.pdf]

Additional experiments

In Experiment S1, we tested the co-efficiency maximization hypothesis under different task instructions. Experiment S2 addressed the hypothesis that co-efficient decisions may have been the result of a direct reciprocity strategy of a partner's efforts: participants performed a non-reciprocal version of the joint task.

Methods

Participants. Thirty right-handed participants took part in Experiment S1, forming dyads. We excluded three pairs from data analysis because the participants had previously known one another. We report the results of 12 dyads (9 mixed-gender and 3 female; N = 24, 9 males, M = 24.5 years, SD = 2.59).

Fifty-four (27 pairs of) right-handed participants took part in Experiment S2. Three pairs were excluded due to misunderstanding instructions, experimenter error and because the co-actors knew each other, respectively. Results for 24 actors from 24 dyads (10 mixed-gender, 12 female; N = 24, 9 males, M = 23 years, SD = 3.41) are reported.

Apparatus. We used the same apparatus as in Experiment 2.

Task, Design and Procedure. The task, design and procedure in Experiments S1 and S2 were identical to Experiment 2, bar the following modifications: in Experiment S1, participants were given a different instruction (i.e. to finish each round as quickly and accurately as possible); in Experiment S2, only one person from each dyad made decisions as Actor 1. In Experiment S1, each participant completed 80 trials, totaling 160 trials for a dyad. In Experiment S2, each session comprised only 80 trials as only one person started the trials in each dyad. Participants in Experiment S1 completed the task on average in M = 16.01 minutes (SD = 6.84) and in Experiment S2, in M = 14.91 minutes (SD = 2.62), while in Experiment 2, dyads took on average 21.49 mins. In Experiment S1, we instructed participants to be fast in addition to being accurate, which is the reason why they were faster

than in Experiment 2. In Experiment S2, the instruction only mentioned accuracy, just like in Experiment 2. Here, one potential explanation for being overall faster may be that in Experiment S2, only one person decided about distributing workload between co-actors. This might have reduced degrees of freedom for overall decision-making, which could have manifested itself in faster completion of the experiment.

Data analyses. Data transformations and analyses were identical to Experiment 2's.

Results

In both experiments, Actor 1 chose sub-paths that maximized the dyad's co-efficiency (Figs. 2c and 2d in the main text). On Congruent trials, actors mostly passed the football over to their partner in the gap closer to themselves (Experiment S1: M = 0.75, SD = 0.30, V = 256, p = .003, r = .71, 95% confidence interval (CI) for the co-efficient choice proportion difference from chance level [arcsine transformed chance level of 0.5 = 0.7854] = [0.97, 1.29]; Experiment S2: M = 0.82, SD = 0.22, V = 287, p < .001, r = .91, 95% CI = [1.07, 1.39]); on Incongruent trials, they chose the more distant gap (Experiment S1: M = 0.92, SD = 0.12, V = 300, p < .001, r = 1.00, 95% CI = [1.28, 1.48]; Experiment S2: M = 0.94, SD = 0.10, V = 300, p < .001, r = 1.00, 95% CI = [1.32, 1.48]). Furthermore, participants made more co-efficient decisions when this entailed facilitating their partner's action by taking the longer sub-path themselves, than when it meant taking the short sub-path (Experiment S1: V = 219, p = .014, r = .46, 95% CI = [0.05, 0.47]; Experiment S2: V = 172, p = .002, r = .15, 95% CI = [0.12, 0.37]). Finally, we also replicated the altruistic bias on Neutral trials: Actor 1 chose the longer sub-path significantly more often than expected by chance (Experiment S1: M = 0.63, SD = 0.28, V = 204.5, p = .044, r = .36, 95% CI = [0.79, 1.10]; Experiment S2: M = 0.63, SD = 0.21, V = 217.5, p = .016, r = .45, 95% CI = [0.82, 1.06]).

To investigate the effect of cost asymmetries between partners on decision-making, we compared co-efficient sub-path choice proportions between each barrier length (Figs. 3c and

3d in the main text). In both experiments, 4 X 2 (Cost Asymmetry X Condition) repeated-measures ANOVAs revealed statistically significant main effects for Cost Asymmetry (Experiment S1: $F(3, 69) = 13.36$, $p < .001$, $\eta^2 = .37$; Experiment S2: Greenhouse-Geisser corrected $F(2.17, 49.86) = 11.00$, $p < .001$, $\eta^2 = .32$) and Condition (Experiment S1: $F(1, 23) = 6.78$, $p = .016$, $\eta^2 = .23$; Experiment S2: $F(1, 23) = 13.69$, $p = .001$, $\eta^2 = .37$), and interactions between Cost Asymmetry and Condition (Experiment S1: Greenhouse-Geisser corrected $F(2.11, 48.48) = 4.00$, $p = .023$, $\eta^2 = .15$; Experiment S2: Greenhouse-Geisser corrected $F(2.07, 47.50) = 3.80$, $p = .028$, $\eta^2 = .14$). Post-hoc Bonferroni-corrected paired-samples t-tests yielded statistically significant effects of Condition on co-efficient choice proportions in trials with 0.75 unit length barriers (Experiment S1: $t(23) = 3.31$, $p = .012$, $d = 0.68$, 95% CI = [0.17, 0.73]; Experiment S2: $t(23) = 3.82$, $p = .004$, $d = 0.78$, 95% CI = [0.13, 0.43]), and in Experiment S2, also in trials with 0 unit length barriers ($t(23) = 3.74$, $p = .004$, $d = 0.76$, 95% CI = [0.12, 0.42]). Participants made more co-efficient choices in Incongruent than in Congruent trials.

Table S1.

Comparisons of speed and accuracy measures between trials where participants made co-efficient and sub-efficient choices

	Co-efficient trials	Sub-efficient trials	Statistic (t)	*df*	*p*	*d*	95% CI for mean difference
Experiment S1							
Collision	N = 24	N = 24	3.34	23	.003*	0.68	[0.14,

	M = 0.28<br>SD = 0.11	M = 0.65<br>SD = 0.53						0.59]
Trial duration (s)	N = 24<br>M = 5.60<br>SD = 0.67	N = 24<br>M = 7.17<br>SD = 1.27	8.94	23	< .001**	1.82	[0.08, 0.13]	
Experiment S2								
Collision	N = 24<br>M = 0.11<br>SD = 0.06	N = 19<br>M = 0.36<br>SD = 0.48	2.31	18	.033*	0.53	[0.02, 0.48]	
Trial duration (s)	N = 24<br>M = 7.46<br>SD = 1.36	N = 19<br>M = 9.68<br>SD = 2.17	11.01	18	< .001**	2.53	[0.09, 0.13]	

*$p < .05$. **$p < .01$.

Co-efficient decisions led to faster and more accurate performance (results of paired-samples t-tests are reported in Table S1).

To explore the effect of practice, we compared co-efficient choice ratios between the first and the second half of each experiment (Block 1 v. Block 2, Experiment S1: 80 trials/block, Experiment S2: 40 trials/block), with paired-samples Wilcoxon signed-rank tests. In both experiments, the ratios of co-efficient choices were significantly higher than chance already in Block 1, regardless of condition (all *p*s < .05). There was a statistically significant increase in the ratio of co-efficient choices between Blocks 1 and 2 in the Congruent condition (Experiment S1: $M_{Block1} = 0.72$, $SD_{Block1} = 0.30$ to $M_{Block2} = 0.80$, $SD_{Block2} = 0.31$, *V*

= 38, *p* = .007, *r* = -.75, 95% CI = [-0.28, -0.07]; Experiment S2: M_{Block1} = 0.76, SD_{Block1} = 0.27 to M_{Block2} = 0.88, SD_{Block2} = 0.19, *V* = 16, *p* = .005, *r* = -.89, 95% CI = [-0.47, -0.12]), as participants selected the co-efficient short path more often in the second than in the first half of the experiment. In Experiment S1, but not in Experiment S2, we also found a significant increase in the ratio of co-efficient choices in the Incongruent condition (Experiment S1: M_{Block1} = 0.88, SD_{Block1} = 0.21 to M_{Block2} = 0.96, SD_{Block2} = 0.10, *V* = 16, *p* = .042, *r* = -.89, 95% CI = [-0.54, -0.01]; Experiment S2: M_{Block1} = 0.93, SD_{Block1} = 0.13 to M_{Block2} = 0.96, SD_{Block2} = 0.08; *V* = 22.5, *p* = .373, *r* = -.85, 95% CI = [-0.35, 0.06]).

Finally, to see whether dyads acted more efficiently than individuals, we compared the ratio of co-efficient choices in Experiments S1 and S2 to the ratio of efficient choices in Experiment 1 (Individual task, see main text). Mann-Whitney U tests with Experiment as a factor found that the ratio of efficient choices in the Congruent condition was statistically significantly higher in Experiment 1 than in Experiment S1 (*U* = 398.5, *p* = .021, *r* = .38, 95% CI for the median difference between the two experiments = [0.00, 0.41]), but the difference was not statistically significantly different between Experiments 1 and S2 (*p* = .179). In the case of the Incongruent conditions, we found that dyads in Experiment S2 chose the co-efficient long sub-paths significantly more often than individuals chose the efficient paths in Experiment 1 (*U* = 194.5, *p* = .047, *r* = -.33, 95% CI = [-0.29, 0.00]), although this difference did not reach statistical significance between Experiments 1 and S1 (*p* = .100). In sum, we found no consistent evidence that dyads made overall more (co-) efficient decisions than the individual participants of Experiment 1.

We calculated the correlation (Spearman's ρ) between liking ratings (Experiment S1: Mdn = 6, SD = 1.28; Experiment S2: Mdn = 6, SD = 1.26) and the arcsine transformed ratios of co-efficient choices, which was not different from zero in either condition (Congruent:

Experiment S1: ρ = -.119, p = .587, Experiment S2: ρ = -.303, p = .151; Incongruent: Experiment S1: ρ = .252, p = .246, Experiment S2: ρ = -.346, p = .097).

Discussion

In Experiment S1, in the experimental conditions, dyads optimized their actions by making decisions that minimized joint costs already from the first half of the task, and in the Neutral condition, we replicated the bias to facilitate a partner's action. We therefore replicated our results under different task instructions, highlighting both the accuracy and speed of performance.

In Experiment S2 we replicated all results of previous joint experiments, indicating that co-efficient decisions are not driven by reciprocity expectations.
